# Dataset from a qualitative survey on Ph.D. entrepreneurship in Italy

**DOI:** 10.1016/j.dib.2018.03.116

**Published:** 2018-03-30

**Authors:** Alessandro Muscio, Laura Ramaciotti

**Affiliations:** a Dipartimento di Scienze Agrarie, degli Alimenti e dell’Ambiente (SAFE), Università di Foggia, Foggia, Italy; b Dipartimento di Economia e Management, Università di Ferrara, Ferrara, Italy

**Keywords:** Ph.D. entrepreneurship, Entrepreneurial University, Start-up, Firm Creation

## Abstract

This article describes questionnaire data on Ph.D. (Doctor of Philosophy) entrepreneurship in Italy. The data includes (i) information recently collected via a questionnaire survey on Ph.D. students; (ii) background information on Italian academic institutions attended by students. We present here some descriptive statistics of the variables included in the dataset. The database includes the responses of 906 students. Students provided information on their employment condition, on their family background and opinions on the Ph.D. course and the institution they attended. Information on regional characteristics and on university policies are also included.

## Specifications Table *[please fill in right-hand column of the table below]*

**Table:** 

Subject area	*Economics*
More specific subject area	*Entrepreneurship, Education*
Type of data	*Cross-section dataset*
How data was acquired	*Qualitative survey, institutional websites, national databanks*
Data format	*Raw*
Experimental factors	–
Experimental features	–
Data source location	https://drive.google.com/file/d/1meBr8dPfLuIemcruz7wghsh1s0YVCZMy/view?usp=sharing
Data accessibility	*Data available with this article and publicly available online here:*https://drive.google.com/file/d/1meBr8dPfLuIemcruz7wghsh1s0YVCZMy/view?usp=sharing

## Value of the data

• While there are some empirical studies on student entrepreneurship and abundant literature on academic entrepreneurship, Ph.D. entrepreneurship is a vastly unexplored phenomenon.
• The database includes original data obtained from the responses to a questionnaire survey administered between 2014 and 2015 on Italian doctorate students. Qualitative data like this is rarely made publicly available.
• The database included original information about students’ perception about their home institution and about their Ph.D. programme.
• The authors welcome future collaborations with other scholars and welcome the opportunity to contribute to the design of a similar survey in other countries.

The data was collected protecting confidentiality and anonymity of the respondents. The dataset provides an original contribution to the understanding of the academic entrepreneurship phenomenon.

## 1 Data

With this survey, the authors intended to explore the entrepreneurial attitude of Ph.D. students, Ph.D. entrepreneurship is a relatively unexplored phenomenon in economics and management [2], [3]. While there is abundant literature on academic entrepreneurship, it focuses almost exclusively on spin-offs start-ups by faculty and staff, largely ignoring the magnitude of the phenomenon of student entrepreneurship [1], [5]. The first results of this line of research have already been published [4].

In order to investigate the factors driving Ph.D. students to start their own venture, the authors created an online questionnaire. The dataset described in this article was obtained from the responses to a questionnaire survey administered between 2014 and 2015 on Italian doctorate students who were supposed to receive the Ph.D. title between 2008 and 2014. Ph.D. students in Italy join 3-year programmes, but they are given the opportunity to extend their studies for one extra year.

The questionnaire was distributed directly by CINECA, an Italian consortium of universities, Research Institutions and the Ministry of Education and Research (MIUR), which holds the contact details of all Italian Ph.D. students and graduates. CINECA sent the questionnaire to around 23,500 individuals and received back 9062 completed questionnaires. We agreed with CINECA to make publicly available 10 per cent of responses to a subset of questionnaire questions. The full database is available upon request to the authors.

The questionnaire asked general questions about their study period, their level of satisfaction with the study programme, their occupational status and entrepreneurial activity. The questionnaire data were complemented by other data sources:

• to control for university level characteristics that might have affected the choice to become an entrepreneur, the survey data were merged with data provided by MIUR on university size, location and research performance;
• to control for the university context, data collected by the Italian National Network for the Valorization of University Research (NETVAL) were merged to the dataset. Variables on the number of spinoffs in 2005–06 in the province where the university is located, on Technology Transfer Offices (TTO) and on their mission, were added to the database;
• data on the availability at the home institution of academic rules supporting start-ups and spin-offs were merged into the dataset. The authors obtained this information from institutional websites;
• data on regional unemployment were drawn from the Italian National Institute of Statistics (ISTAT) databases.

The dataset is organized in a cross-sectional format, including student responses to a set of qualitative and quantitative questions and some general information on the university they attended and its geographical location, obtained from other sources. The dataset is available in “.dta” format, compatible with the statistical software STATA14^©^. Labels describe the data measured with the 86 variables included.

## 2 Experimental design, materials and methods

The data shows that at the time of the survey 61% of respondents completed their Ph.D. studies.

**Fig. 1:**
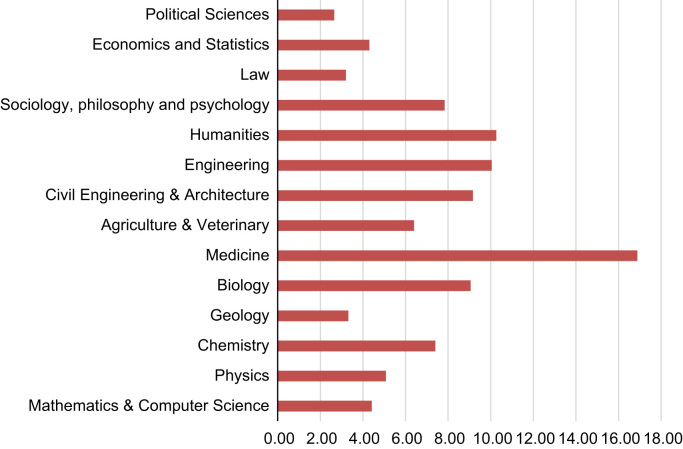
Ph.D. subject area (per cent).

The majority of students studied Medical Studies (Fig. 1), followed by Humanities and Engineering.

On average students were born in 1980, 50% of them are male and 2% of them is of non-Italian nationality. For what concerns students’ background, at least one of their parents hold a university degree in 37% of cases, in 6% of cases one of them is a university lecturer and in 9% of cases one of them is an entrepreneur.

**Fig. 2:**
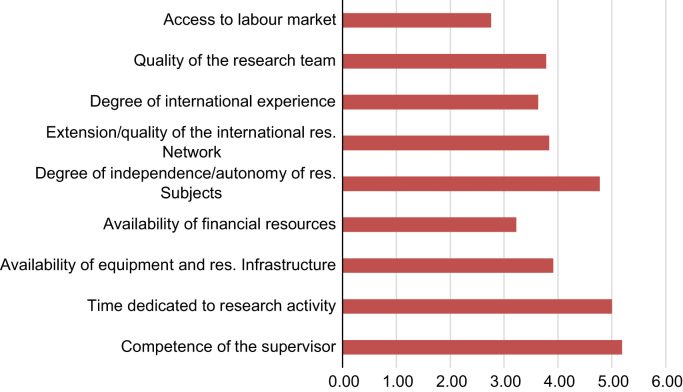
Students’ opinion on the Ph.D. programme (1–6 = highest).

Overall students rated sufficiently well the Ph.D. programme on a Likert scale ranging from 1 (lowest) to 6 (highest) (Fig. 2). The lowest score was attributed to the access to the labour market after the Ph.D., while the highest score was given to the freedom given to the student in the choice of the research subject, to the time made available to research activity and to the competence of the supervisor.

Ph.D. students in Italy can access Ph.D. programmes with or without government scholarships. In 12% of cases students earned a scholarship funded by the private sector and in 18% of cases Ph.D. research was based on collaboration with private companies. Overall students claimed that they dedicated 75% of their time to basic research.

**Fig. 3:**
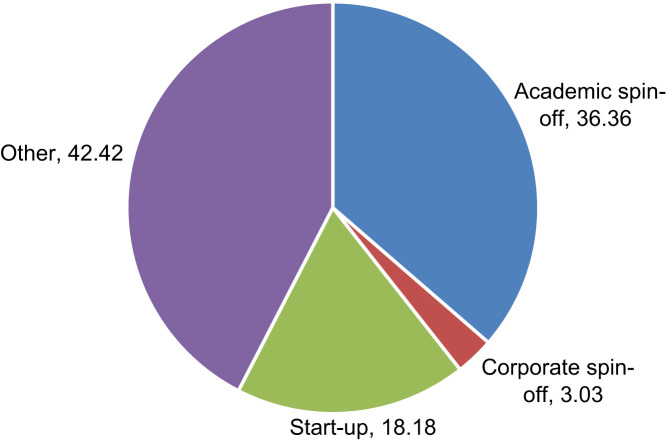
Type of start-up established by the student.

73% of students was employed at the time of the survey: 62% worked in academia and 10% was an autonomous worker. 7% of students attended to courses on entrepreneurship during the Ph.D. programme. Overall, 6% of students claimed that she/he established or was involved in the establishment of a business start-up and 5% of students claimed that they established or were involved in the establishment of a business start-up that was still active at the time of the survey. 45% of students abandoned the idea of establishing a start-up. On average, those start-ups created by students that are still active employ 6 employees and were funded some 4.6 years before the survey. Fig. 2 presents information on the type of business established (Fig. 3).

**Fig. 4:**
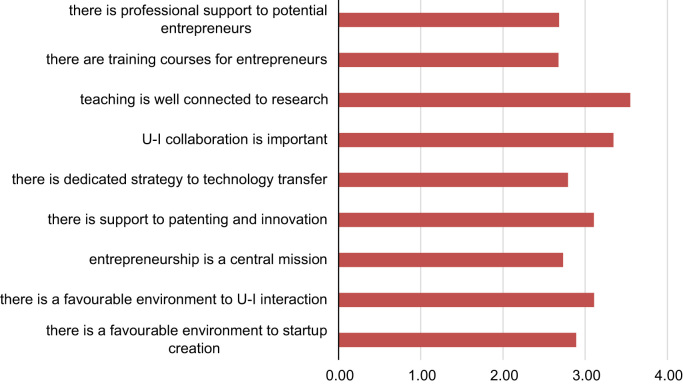
The student agrees that in the home university (1–6 = highest):.

Fig. 4 presents students’ opinion concerning several aspects regarding research and entrepreneurial activities at their home university. On a Likert scale ranging from 1 (lowest) to 6 (highest), students ranked highest the degree to which teaching is connected to research activity, the importance of university-industry collaboration, support to patenting and the presence of a favourable environment to university-industry collaboration.

The last set of variables control for the context where the students attended to their Ph.D. course.

University size is expressed in terms of number of students: 1 small (< 10,000); 2 medium (10,000–15,000); 3 large (15,000–40,000); 4 mega (> 40,000). 4.3% of students attended small universities, 9.1% attended medium sized universities, 37.8% attended large universities and the remaining 48.6% studied in mega universities. 80% of academic institutions hosts a medical school and in 5% of cases students attended a polytechnic university (four in Italy). in 98% of cases students attended public universities.

The database includes information on university research rating published by MIUR in 2014, based on evaluation of the research output carried out over the period 2004–10. This composite indicator accounts for peer review evaluations of research activity carried out at academic institutions (patents, impact factor of journal articles, etc.). The estimated values for the index range from 0 to 1.84, with a mean value of 1.00.

Academic institutions were located in 39% of cases in northern Italy, in 29% of cases in central Italy and 32% of cases in Southern Italy. The unemployment rate in the administrative province where the university is located was 7.2% in 2006. On average, there were 2.4 spinoffs in the area where the university is located in the period 2005–06.

Finally, 86% of institutions had a TTO in 2006, whose mission was in 92% of cases to support entrepreneurship. In 65% of cases academic institutions adopted dedicated rules on spinoff creation.
